# Extracorporeal membrane oxygenation programs for COVID-19 in China

**DOI:** 10.1186/s13054-020-03047-6

**Published:** 2020-06-08

**Authors:** Chenglong Li, Xiaotong Hou, Zhaohui Tong, Haibo Qiu, Yimin Li, Ang Li, Chinese Society of Extracorporeal Life Support (CSECLS)

**Affiliations:** 1 Center for Cardiac Intensive Care, Beijing Anzhen Hospital, Capital Medical University, 2 Anzhen Rd, Chaoyang District, Beijing, 100029 China; 2 Department of Respiratory and Critical Care Medicine, Beijing Institute of Respiratory Medicine, Beijing Chao-yang Hospital, Capital Medical University, Beijing, China; 3 Department of Critical Care Medicine, Zhongda Hospital, School of Medicine, Southeast University, Jiangsu, China; 4 The State Key Laboratory of Respiratory Diseases, Guangzhou Institute of Respiratory Health, The First Affiliated Hospital of Guangzhou Medical University, Guangdong, China; 5 Department of Critical Care Medicine, Beijing Ditan Hospital, Capital Medical University, Beijing, China

Dear Editor,

The outbreak of coronavirus disease (COVID-19) was first reported in Wuhan, the capital city of Hubei province, and may lead to severe pneumonia and acute respiratory distress syndrome (ARDS). Extracorporeal membrane oxygenation (ECMO), as a temporary life support technique for refractory respiratory or cardiac failure, has been applied in COVID-19 patients [1]. However, the impact of ECMO on outcomes from COVID-19 was controversial. Referring to the present case series and the COVID-19 cohort in China, the mortality of patients undergoing ECMO ranged from 42 to 83% [2, 3]. The Chinese Society of Extracorporeal Life Support (CSECLS) performed a survey of ECMO programs for COVID-19 in China, aimed at investigating the program organization and the potential factors associated with outcomes during the pandemic.

This voluntary survey was disseminated via e-mail and WeChat to 365 ECMO programs registered with the CSECLS on March 23, 2020. Through March 29, we had received 350 individual responses from 270 (74.0%) ECMO programs in total. When analyzing program characteristics, the program directors’ or coordinators’ responses were adopted.

One hundred eleven individual responses from 79 ECMO programs (30 in Hubei and 49 outside Hubei) applied ECMO in patients with COVID-19 pneumonia and ARDS were analyzed. Respondents included those located in 25 provinces within China before COVID-19 outbroke. Twenty-seven respondents belonged to the medical assistance teams which were dispatched to aid Hubei and 39 respondents aided other hospitals in their original province, while 45 respondents managed COVID-19 patients with ECMO within their original hospitals. Fifty-one of 79 ECMO programs (15 in Hubei and 36 outside Hubei) were organized temporarily in response to the crisis. Thirty-two hospitals with temporary ECMO programs did not have any ECMO cases before. The geographic distribution of the 79 ECMO programs and responders’ aid to Hubei is shown in Fig. 1. Patient management characteristics are illustrated in Table 1. Compared with ECMO programs in Hubei, more programs outside Hubei initiated ECMO in older patients (36.7% vs 3.3% in age ≥ 75, *p* = 0.001; 55.1% vs 26.7% in age 65–74, *p* = 0.014).

**Fig. 1:**
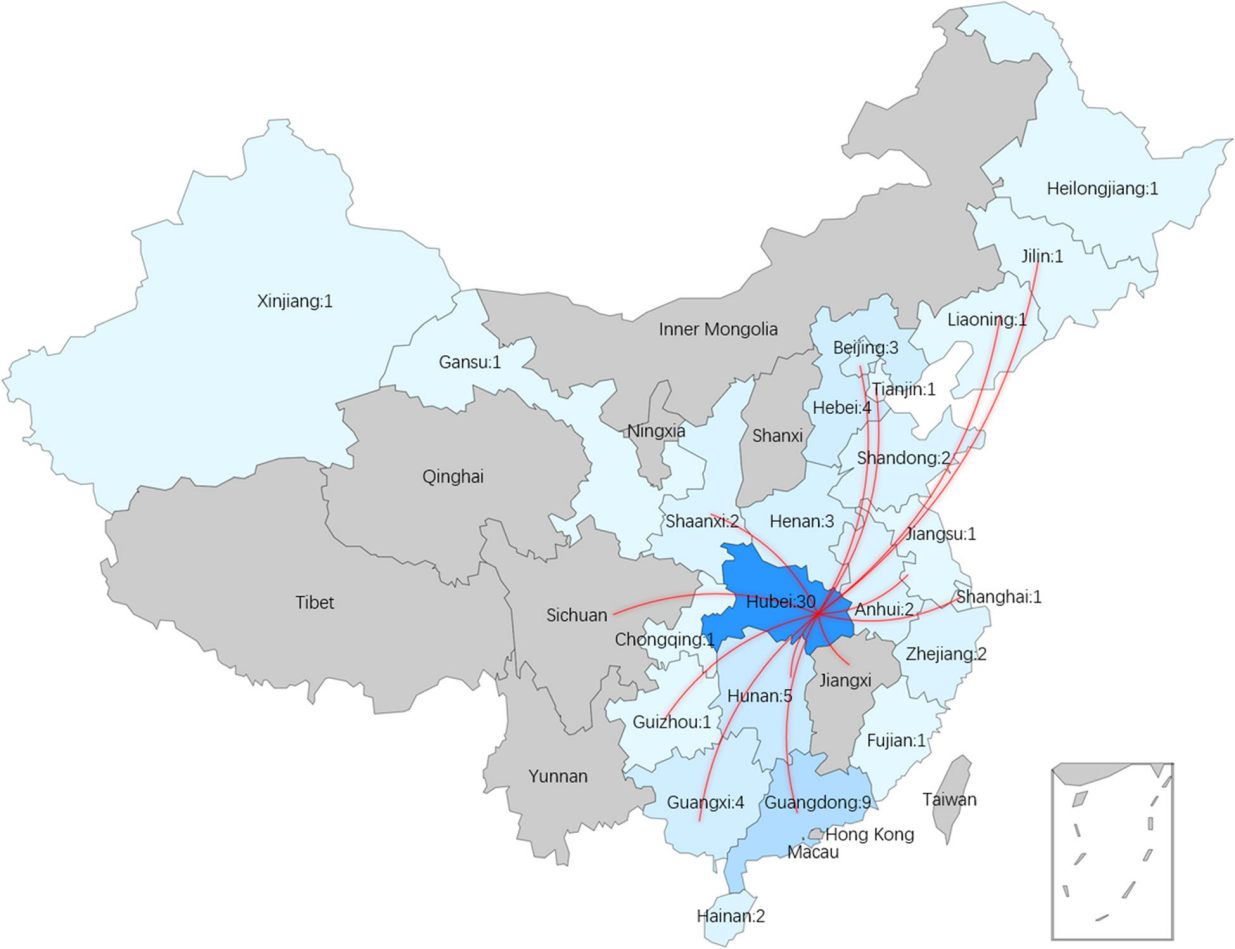
The geography distribution of ECMO programs for COVID-19 in China. The total 79 ECMO programs for COVID-19 by province (with the number of ECMO programs in each province) and respondents’ aid to Hubei from other provinces (red lines) were shown

**Table 1:** Patient management responded by ECMO programs

Question	Total ECMO programs, *n* = 79 [*n* (%)]
ECMO cases for COVID-19	
≤ 5	68 (86.1%)
> 5	11 (13.9%)
The source of ECMO patients for COVID-19	
In-hospital patient	62 (78.5%)
Transferred from other hospitals, then initiated ECMO	24 (30.4%)
Initiated ECMO in other hospitals, then transferred	8 (10.1%)
Initiated ECMO in COVID-19 patients whose age	
< 65	54 (68.4%)
65–74	35 (44.3%)
≥ 75	19 (24.1%)
Therapy before ECMO initiation	
Recruitment maneuvers	65 (82.3%)
Neuromuscular blockers	64 (81.0%)
Prone position	61 (77.2%)
Therapy after ECMO initiation	
Recruitment maneuvers	56 (70.9%)
Neuromuscular blockers	61 (77.2%)
Prone position	61 (77.2%)
Bronchoscopy	59 (74.7%)
CT scan	40 (50.6%)

*ECMO* extracorporeal membrane oxygenation, *CT* computer tomography

Our findings provide evidence of the current condition of ECMO programs for COVID-19 across China. Fifty-one ECMO programs were newly organized. It was most efficient to rearrange medical workers and resources rather than starting new ECMO programs amid the crisis, given that inexperienced ECMO programs and hospitals might lead to unfavorable outcomes. Since ECMO is a complicated and high-risk therapy, adequate training and high-volume experience are indispensable [4].

We also found a difference in age between ECMO patients in Hubei and outside Hubei. Seventy-five percent of COVID-19 cases in China were diagnosed in Hubei [5]. With limited medical resources in Hubei, patients with a higher likelihood of survival were chosen to receive ECMO, namely younger patients. However, medical resources were adequate outside Hubei. That might be the main reason for more ECMO programs outside Hubei applied ECMO in older patients (age > 65), aiming at minimizing the local mortality of COVID-19. Age is a key driver of mortality, helping clinicians to select the most appropriate candidates for ECMO among severe ARDS patients [6]. However, age should be reconsidered in the discussions of indications for ECMO in COVID-19. To the limitation, the patient’s detailed characteristic was not obtained in the present study. Further multicenter registry on COVID-19 patients receiving ECMO support would be performed.

To summarize, our large national survey provided detailed information regarding the organization of ECMO programs for COVID-19 in China. To improve outcomes with ECMO during the pandemic, it is key to provide information about ECMO experience, patient selection, and resource allocation to ECMO programs throughout the world.

## Data Availability

The datasets used in the present study are available from the first author and corresponding authors on reasonable request.

## Abbreviations

COVID-19: Coronavirus disease 2019
CSECLS: Chinese Society of Extracorporeal Life Support
CT: Computer tomography
